# Supplementary figures and images for: Observer: creation of a novel multimodal dataset for outpatient care research
Source: J Am Med Inform Assoc. 2025 Oct 27;33(2):424–33. doi: 10.1093/jamia/ocaf182 (PMC12844583; doi:10.1093/jamia/ocaf182)

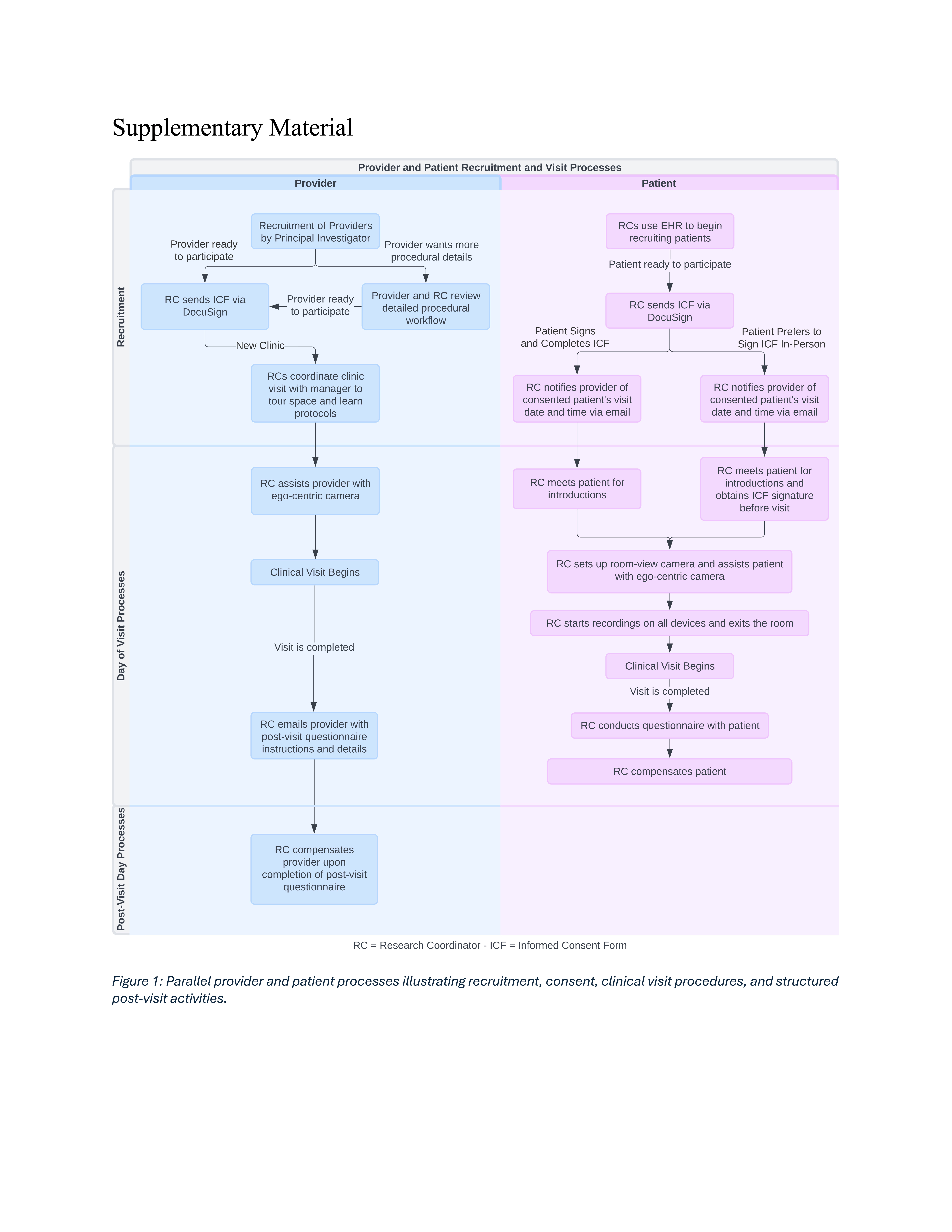

Supplement: ocaf182_Supplementary_Data [file ocaf182_supplementary_data.zip › ocaf182_Supplementary_Data/Supplemental_Figure_1.jpg]
